# Anticancer Effect and Structure-Activity Analysis of Marine Products Isolated from Metabolites of Mangrove Fungi in the South China Sea

**DOI:** 10.3390/md8041094

**Published:** 2010-04-01

**Authors:** Li-yang Tao, Jian-ye Zhang, Yong-ju Liang, Li-ming Chen, Li-sheng Zhen, Fang Wang, Yan-jun Mi, Zhi-gang She, Kenneth Kin Wah To, Yong-cheng Lin, Li-wu Fu

**Affiliations:** 1 State Key Laboratory for Oncology in South China, Cancer Center, Sun Yat-Sen University, Guangzhou, 510060, China; E-Mails: sohutly@163.com (L.T.); jianyez2003@yahoo.com.cn (J.Z.); liangyju@mail.sysu.edu.cn (Y.L.); chlm78@yahoo.com.cn (L.C.); lesley860221@163.com (L.Z.); wangfang0203@163.com (F.W.); myjgj_77@163.com (Y.M.); 2 School of Pharmacy, The Chinese University of Hong Kong, Hong Kong, China; E-Mail: kennethto@cuhk.edu.hk (K.K.W.T.); 3 School of Chemistry and Chemical Engineering, Sun Yat-Sen University, Guangzhou, 510275, China

**Keywords:** mangrove fungi, multidrug resistance (MDR), quinones, xyloketal, isoflavone, prostaglandins

## Abstract

Marine-derived fungi provide plenty of structurally unique and biologically active secondary metabolites. We screened 87 marine products from mangrove fungi in the South China Sea for anticancer activity by MTT assay. 14% of the compounds (11/86) exhibited a potent activity against cancer *in vitro*. Importantly, some compounds such as compounds 78 and 81 appeared to be promising for treating cancer patients with multidrug resistance, which should encourage more efforts to isolate promising candidates for further development as clinically useful chemotherapeutic drugs. Furthermore, DNA intercalation was not involved in their anticancer activities, as determined by DNA binding assay. On the other hand, the structure-activity analysis indicated that the hydroxyl group was important for their cytotoxic activity and that bulky functional groups such as phenyl rings could result in a loss of biological activity, which will direct the further development of marine product-based derivatives.

## 1. Introduction

The majority of species are found in the oceans, which constitute 70% of the world’s surface. Marine organisms are a rich source of chemical products [1,2]. In recent years, a renaissance has occurred in marine pharmacology. Complex and highly chiral structures have been optimized by high salt concentrations and high pressure environments over millions of years, which confers marine organisms the potential to produce valuable therapeutic entities [3,4]. Emerging evidence suggests that marine natural products, especially the secondary metabolites from marine organisms, are far more likely to yield anticancer drugs than terrestrial sources [1,5]. For example, Arc-C (cytarabine, an antileukemic drug) and trabectedin (Yondelis, ET-743, an agent for treating soft tissue sarcoma) were developed from marine sources [3,6]. Mangroves, a kind of special host plants, are a resource of abundant endophytic fungi. Marine-derived fungi have proven to be a rich source of structurally unique and biologically active secondary metabolites [7]. In the last decade, there has been a dramatic increase in the number of preclinical anticancer lead compounds extracted from metabolites of marine-derived fungi [8–10].

Chemotherapy is currently the primary treatment modality in many tumors. However, the development of multidrug resistance (MDR) to chemotherapeutic drugs is a main obstacle for the successful treatment of malignant tumors. One of the best known mechanisms of MDR is the overexpression of ATP-binding cassette (ABC) transporters that actively pump drugs out of tumor cells [11,12]. Therefore, the development of novel chemotherapeutic agents would play a key role in the treatment of refractory or relapsing cancer patients. Nowadays, the chemical, biological and ecological diversity of the marine ecosystem has contributed immensely potent antitumor compounds [13]. It is believed that a rich source of anticancer drug candidates could be obtained from marine organisms or their metabolites. In this paper, we describe qualitative structure-activity relations for a number of mangrove-derived secondary metabolites. Some further compounds have not been obtained from nature and will be described in detail later elsewhere.

## 2. Results and Discussion

Eighty-six compounds isolated and identified from metabolites of mangrove fungi in the South China Sea were screened for activity inhibiting cancer cell growth by MTT assay. Eleven of them showed potent cytotoxic activity in KB, KBv200, MCF-7, MCF-7/adr, and A549 cells, with IC_50_ values less than 50 μmol/L. These are the compounds 1, 4, 7, 8, 9, 11, 13, 14, 18, 78 and 81. Importantly, compounds 78 and 81 exhibited similar IC_50_ values in both ABCB1/P-glycoprotein (P-gp) overexpressing MDR cells such as KBv200, MCF-7/adr and their parental sensitive cells such as KB, MCF-7 (Table 1). These results suggest that we found some lead compounds from marine production and some are promising to treat MDR cancer patients.

To understand whether the compounds are specially targeting the cancer cells rather than normal cells, we compared the effect of the compounds on inhibiting cell growth in cancer cells and normal cells. The results showed cancer cells were more sensitive to compounds 1, 18, 78 and 81 than the normal liver LO2 cells. However, compound 4 and 11 also exhibited growth inhibitory activity in LO2 cells. Of course, the toxicity of the compounds needs further study.

The core structure of compounds 7–10 is a xanthone, which is a main constituent in the mangosteen fruit and was firstly isolated from marine products. Xanthone has potent antioxidant and anticancer activities and regulates the function of immunity [14]. Compounds 11–19 are anthraquinones, which have been demonstrated to exert various physiological activities such as antibiotic and anticancer properties [10,15,16]. Similar to doxorubicin, compound 7, 9, 13, 14, exhibited much higher IC_50_ values in MDR cells such as KBv200 cells and MCF-7/adr cells than in parental sensitive cells such as KB cells and MCF-7 cells (Table 1). This suggests that MDR cells are extremely resistant to compound 7, 9, 13, 14, which may be substrates of the multidrug resistant transporter ABCB1/P-glycoprotein.

**Figure 1:**
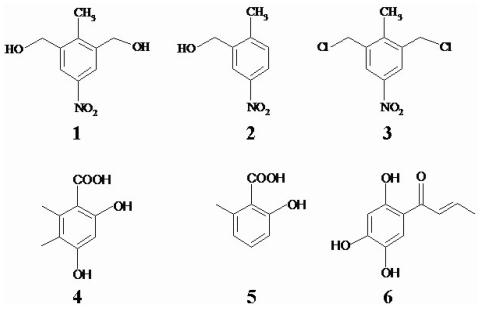
The chemical structures of six multi-substituent phenyl derivatives.

The analysis of structure-activity is important to direct further synthesis. Compared with compound 1, compounds 2, 3 and 5 lack one or two hydroxyl groups, whereas compound 6 has an altered position of the hydroxyl group, which apparently is responsible for loss of cytotoxic activity among six multi-substituent phenyl derivatives (Figure 1). However, the cytotoxic effect was maintained in compound 4 in which the nitro-substituent was absent. This suggests that the para-positioned hydroxyl group is necessary in these compounds to inhibit cancer cells growth.

**Figure 2:**
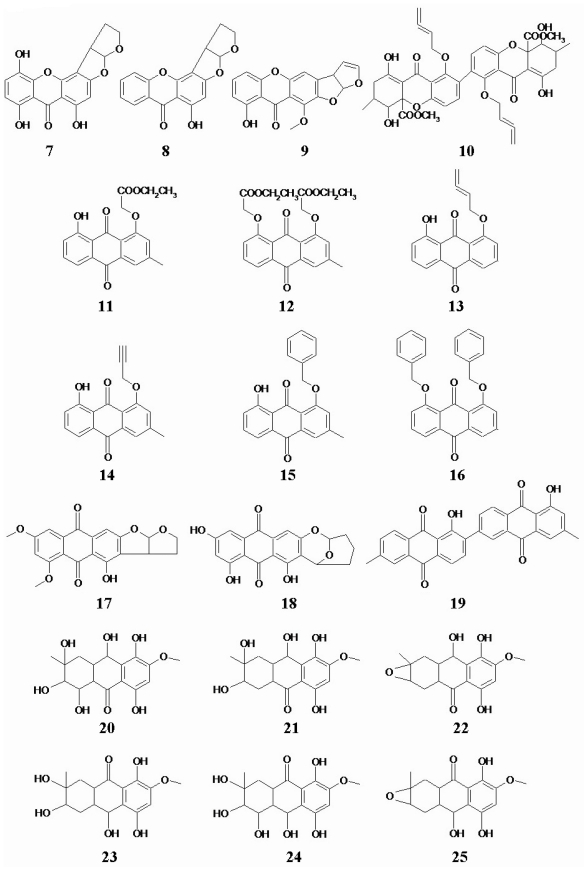
Chemical structures of 19 quinone analogs.

The 5-hydroxyl group was critical to the antitumor activity in quinone derivatives, whereas a phenyl ring was found to result in a loss of activity (Figure 2, Table 1) among compound 7–25. On the other hand, the tetracycline analogs, compounds 20–25, were found to be devoid of any cytotoxic activity.

**Figure 3:**
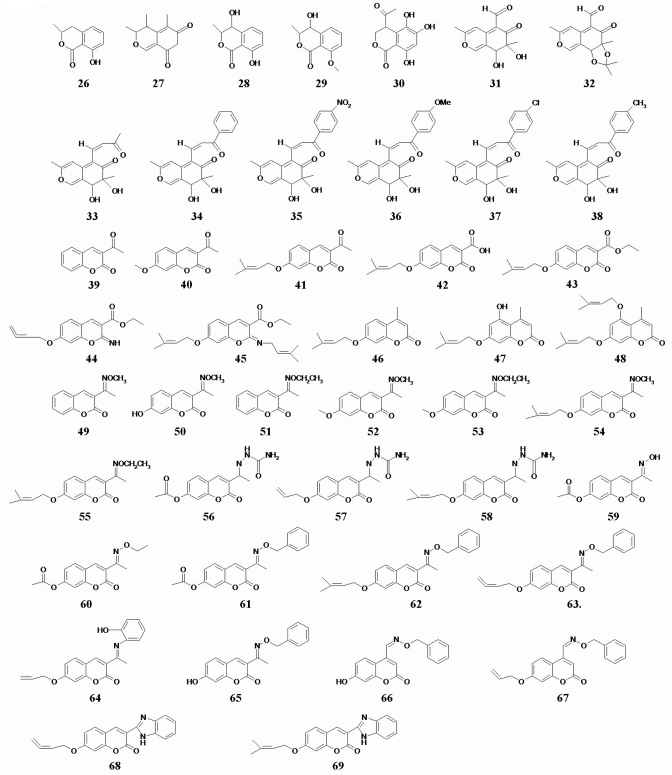
Chemical structures of 44 coumarin analogs.

The coumarin was the precursor for several anticoagulants [17]. The coumarin derivatives (compound 26–69) did not demonstrate significant cytotoxic activity (IC_50_ > 50 μmol/L). Their chemical structures are shown in Figure 3.

**Figure 4:**
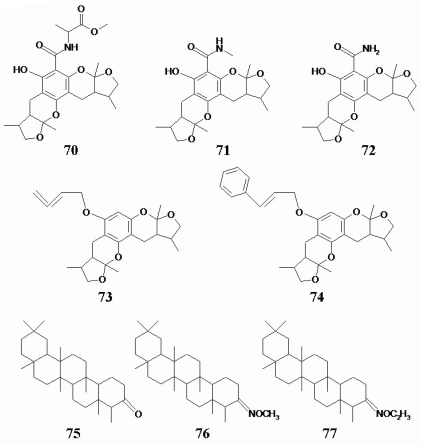
The chemical structures of eight condensed ring compounds.

The compounds 70–74 belong to xyloketal analogs, condensed ring compounds [18,19]. No appreciable antitumor activity was found for these eight compounds (IC_50_ > 50 μmol/L). Their chemical structures are exhibited in Figure 4.

**Figure 5:**
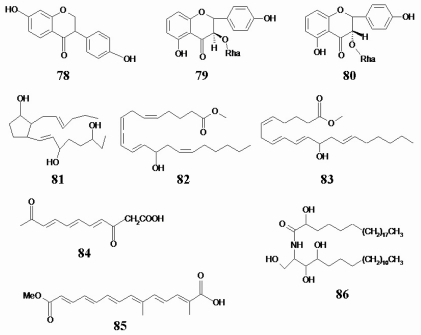
Chemical structures of three isoflavonoid analogs and six fatty acid derivatives.

The compound 78 is an isoflavone analog, which was reported to have a preventive effect against various cancers [20,21]. It exhibited a cytotoxicity targeted to cancer cells. However, its analogs, the compounds 79 and 80, were not found to inhibit cell growth in cancer cells (IC_50_ > 50 μmol/L). The compound 81, a prostaglandin analog and fatty acid derivative, showed potent anticancer activity *in vitro* by MTT assay. However, compounds 82–86, fatty acid derivatives with similar structure to compound 81, did not show anticancer activity (Table 1). The structures of the compounds 78–86 are shown in Figure 5.

**Figure 6:**
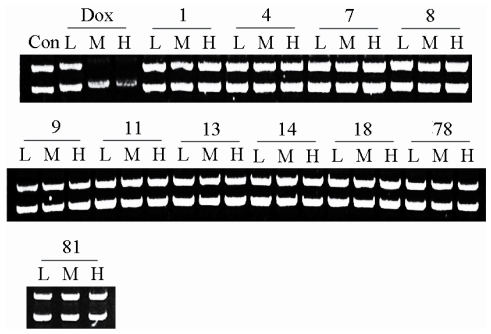
The ability of 11 compounds with IC_50_ value less 50μmol/L to intercalate into DNA. A representative gel image after electrophoresis of the treated DNA vector on 1% agarose for 40 min. The results show that doxorubicin (Dox), a positive control, effectively intercalated to DNA in concentration-dependent manner. However, no compound showed DNA binding in the concentrations of 10 (L), 100 (M) or 200 (H) μmol/L.

As we know, the intercalation of DNA is an important mechanism for several chemotherapeutical drugs such as doxorubicin and cisplatin, and many others [10]. Therefore, we examined the ability of the compounds whose IC_50_ value was less than 50 μmol/L to intercalate into DNA. Compounds 1, 4, 7–9, 11, 13, 14, 18, 78 and 81 were included. The results show that doxorubicin as a standard effectively intercalated into DNA in a concentration-dependent manner. However, none of the compounds investigated here showed binding to DNA even at a very high concentration (Figure 6). These results indicated that DNA intercalation is not involved in the cytotoxic effect induced by these compounds.

In conclusion, we screened 86 marine products for anticancer activity by MTT assay. There were 14% (11/86) compounds that exhibited a potent activity against cancer *in vitro*. The analysis of structure-activity will direct the further development of marine product-based derivatives. Importantly, some compounds, such as compound 78 and 81, appeared to be promising in treating MDR cancer patients.

## 3. Experimental Section

### 3.1. Chemicals and reagents

Doxorubicin (DOX), 3-(4,5-dimethylthiazol-yl)-2,5-diphenyltetrazolium bromide (MTT) and other chemicals were obtained from Sigma Chemical Co. (San Diego, CA, USA). Dulbecco’s modified Eagle’s medium (DMEM) and RPMI 1640 were products of Gibco BRL.

### 3.2. Isolation of compounds from metabolites of marine-derived fungi

Marine-derived fungi were isolated from the mangroves of the South China Sea. The fungi numbers were Phomopsis sp. ZSU-H76 [22], Phomopsis sp. ZZF08 [23], Paecilomyces sp. (tree 1–7) [24], Sargassum sp. ZZF36 [25], Halorosellinia sp. No. 1403 [26], SBE-14 [ 27] and B60 [28]. All compounds were isolated by She Zhigang, a professor at Sun Yat-Sen University. The structures were elucidated by comprehensive spectral analysis, including 2D NMR spectroscopy.

### 3.3. Tumor cell culture

The cell lines were cultured in DMEM or RPMI 1640 containing 10% fetal bovine serum at 37 °C in the presence of 5% CO_2_. The cell lines were the oral epidermoid carcinoma cell lines KB and its vincristine-selected ABCB1-overexpressing KBv200 [10]; the breast carcinoma cell lines MCF-7 and its doxorubicin-selected ABCB1-overexpressing MCF-7/adr [29]; the lung carcinoma cell line A549 and normal liver cell line LO2.

### 3.4. Anticancer activity assays

The MTT assay was used to evaluate the cytotoxicity of a number of compounds previously isolated from mangrove-derived fungi as described [30,31]. Briefly, 3–4 × 10^3^ cells were incubated in 96-well plates and allowed to attach overnight before compounds at full range concentrations were added. The concentrations were chosen so that the highest concentration killed most of the cells and the lowest killed none of the cells. After 72 h treatment, 0.5 mg/ml MTT was added to each well for an additional 4 h. Subsequently, the supernatant was removed, and the purple MTT-formazan crystals were dissolved by DMSO. Finally, absorbance was recorded at 540 nm with 655 nm as a reference filter using a Model 550 Microplate Reader (Bio-Rad, USA). Experiments were performed at least three times. The standard chemotherapeutic agent doxorubicin was used as a positive control in MTT assay and normal liver cell line LO2 was used to show whether cytotoxic activity was tumor-specific. The concentrations required to inhibit growth by 50% (IC_50_) were calculated from survival curves using the Bliss method.

### 3.5. DNA binding assay

10, 100 or 200 μmol/L compounds were incubated with 1 μg pcDNA3.1(+) at 37 °C for 3 h. The mixtures were then subjected to electrophoresis on a 1% agarose gel, pre-stained with 0.5 μg/ml ethidium bromide, at 60 V for 40 min. Doxorubicin (DOX) was used as a positive control.

## Figures and Tables

**Table 1:** Cytotoxic effect of marine products in human cancer cells and a normal liver LO2 cells.

Compound	IC_50_ value (μmol/L)					
	KB	KBv200	MCF-7	MCF-7/adr	A549	LO2
Doxorubicin	0.05 ± 0.003	3.21 ± 0.12	0.39 ± 0.02	22.33 ± 1.56	1.67 ± 0.09	0.11 ± 0.01

Multi-substituent phenyl derivatives						
**1**	1.57 ± 0.08	2.67 ± 0.17	1.38 ± 0.07	2.34 ± 0.11	2.71 ± 0.15	6.59 ± 0.44
**2**	>50	>50	>50	>50	>50	>50
**3**	>50	>50	>50	>50	>50	>50
**4**	1.15 ± 0.06	6.74 ± 0.38	11.74 ± 0.86	35.67 ± 2.08	17.10 ± 1.03	16.48 ± 1.22
**5**	>50	>50	>50	>50	>50	>50
**6**	>50	>50	>50	>50	>50	>50
Mangrove-derived quinones						
**7**	0.03 ± 0.001	9.08 ± 0.65	0.17 ± 0.01	31.56 ± 1.83	16.51 ± 0.88	47.35 ± 2.04
**8**	0.71 ± 0.03	17.20 ± 0.96	2.53 ± 0.14	9.37 ± 0.42	>50	>50
**9**	0.94 ± 0.01	47.98 ± 3.41	1.43 ± 0.09	31.60 ± 1.36	>50	>50
**10**	>50	>50	>50	>50	>50	>50
**11**	5.89 ± 0.37	18.94 ± 1.20	13.23 ± 0.84	35.34 ± 2.77	21.34 ± 1.60	28.88 ± 1.46
**12**	>50	>50	>50	>50	>50	>50
**13**	21.00 ± 1.35	>50	16.32 ± 0.71	>50	31.23 ± 2.02	>50
**14**	11.86 ± 0.69	>50	35.23 ± 1.87	>50	23.53 ± 1.32	37.51 ± 1.64
**15**	>50	>50	>50	>50	>50	>50
**16**	>50	>50	>50	>50	>50	>50
**17**	>50	>50	>50	>50	>50	>50
**18**	28.19 ± 1.66	44.63 ± 2.57	17.22 ± 0.95	24.96 ± 1.06	33.89 ± 2.31	>50
**19**	>50	>50	>50	>50	>50	>50
**20**	>50	>50	>50	>50	>50	>50
**21**	>50	>50	>50	>50	>50	>50
**22**	>50	>50	>50	>50	>50	>50
**23**	>50	>50	>50	>50	>50	>50
**24**	>50	>50	>50	>50	>50	>50
**25**	>50	>50	>50	>50	>50	>50
Isoflavone analogs						
**78**	8.63 ± 0.57	9.37 ± 0.61	19.77 ± 0.89	24.95 ± 1.15	14.88 ± 0.64	33.62 ± 2.06
**79**	>50	>50	>50	>50	>50	>50
**80**	>50	>50	>50	>50	>50	>50
Fatty acid derivatives						
**81**	0.37 ± 0.01	0.39 ± 0.02	0.41 ± 0.02	0.49 ± 0.02	0.34 ± 0.01	0.72 ± 0.03
**82**	>50	>50	>50	>50	>50	>50
**83**	>50	>50	>50	>50	>50	>50
**84**	>50	>50	>50	>50	>50	>50
**85**	>50	>50	>50	>50	>50	>50
**86**	>50	>50	>50	>50	>50	>50
